# A study of demographic and clinical characteristics of consumers of non-steroidal anti-inflammatory drugs: a large population-based study utilizing enrollment phase data from the tabari cohort

**DOI:** 10.1186/s12889-025-24005-3

**Published:** 2025-08-27

**Authors:** Amirhossein Hessami, Ava Pourali, Majid Saeedi, Ali Asghar Nadi Ghara, Motahareh Kheradmand, Mahmood Moosazadeh

**Affiliations:** 1 Non-Communicable Disease Institute, Mazandaran University of Medical Sciences, Sari, Iran; 2 Department of Pharmaceutics, Faculty of Pharmacy, Mazandaran University of Medical Sciences, Sari, Iran; 3 Health Science Research Center, Mazandaran University of Medical Sciences, Sari, Iran; 4 Gastrointestinal Cancer Research Center, Non-Communicable Disease Institute, Mazandaran University of Medical Sciences, Sari, Iran

**Keywords:** Non-steroidal anti-inflammatory drugs, Pain, Demographic factors, Comorbidity

## Abstract

**Background:**

Non-steroidal anti-inflammatory drugs (NSAIDs) are widely used for managing pain and inflammatory conditions. However, their usage patterns and associated risk factors in the general population remain insufficiently understood, particularly in the Iranian context. This study aims to explore the epidemiological and clinical factors associated with NSAID consumption in a population-based cohort.

**Methods:**

In this cross-sectional study, data from the enrollment phase of the Tabari Cohort Study, encompassing 9,939 participants aged 35–70 years, were analyzed. NSAIDs usage was assessed based on self-reported consumption for at least 30 days. Logistic regression models were employed to identify demographic, behavioral, and clinical predictors of NSAID use, adjusting for potential confounders.

**Results:**

The prevalence of NSAIDs use was 14.7%, increasing with age, obesity, and the number of comorbidities. Multivariable analysis identified significant predictors, including older age (OR: 5.01 for 60–70 years), obesity (OR: 1.40), urban residence (OR: 1.71), and substance use (OR: 1.34). Comorbid conditions, such as diabetes, hypertension, and coronary heart disease, showed strong associations, with NSAIDs use increasing markedly as the number of chronic conditions rose (OR: 36.35 for seven or more comorbidities, P for trend < 0.001).

**Conclusion:**

This study highlights the high prevalence of NSAIDs use and its association with demographic and clinical factors. The findings underscore the need for careful monitoring of NSAID consumption, particularly among individuals with multiple comorbidities, to mitigate risks and optimize pain management strategies.

**Graphical abstract:**

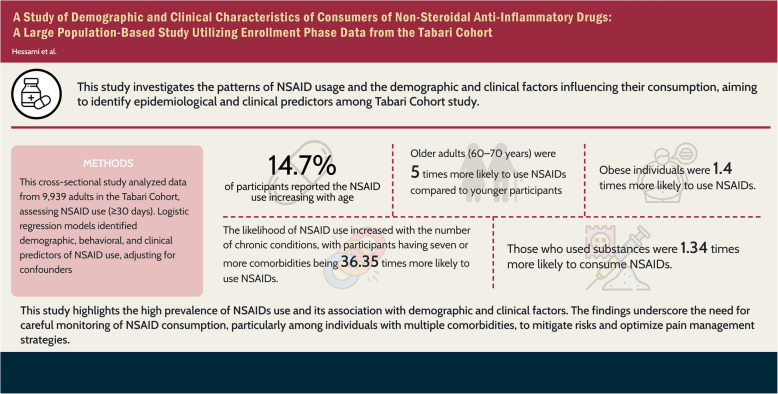

## Background

Non-steroidal anti-inflammatory drugs (NSAIDs) are commonly prescribed for various conditions, including the short-term and long-term management of pain, trauma, and inflammatory diseases, as well as for elderly individuals with chronic pain [1–4]. NSAIDs exert their effects by inhibiting the cyclooxygenase enzyme (COX), which converts arachidonic acid into thromboxanes, prostaglandins, and prostacyclins. There are two cyclooxygenase isoenzymes: COX-1 and COX-2. COX-1 plays a crucial role in maintaining the gastrointestinal mucosa lining, kidney function, and platelet aggregation, whereas COX-2 is predominantly expressed during inflammatory responses. Based on these mechanisms, NSAIDs are categorized as either non-selective (inhibiting both COX-1 and COX-2) such as Ibuprofen, Naproxen and Mefenamic acid or COX-2 selective such as Celecoxib [5].

Previous literature indicated that NSAIDs usage has increased significantly over time [6]. It is estimated that over 1 billion NSAIDs prescriptions are issued annually worldwide, with approximately 30 million people using these drugs daily [7].

Although NSAIDs are generally considered safe and effective, adverse drug events, particularly with less selective COX inhibitors, have been widely reported. These adverse effects include gastrointestinal bleeding [8], hepatic disorders [9], acute myocardial infarction [10], elevated blood pressure [11], and acute renal failure [12]. Moreover, NSAIDs consumption and associated side effects vary according to age, gender, ethnicity, and the presence of comorbidities [1, 13]. Understanding the demographic and clinical factors associated with NSAID use is essential, as these characteristics are also linked to an increased risk of adverse outcomes particularly in high-risk populations [1]. Identifying these predictors can guide safer prescribing and targeted interventions in vulnerable groups.

A population-based study conducted in Spain (MCC-Spain) identified significant gender disparities in the use of NSAIDs. Non-aspirin NSAIDs were found to be more frequently used by women compared to men (38.8% vs. 22.3%), while aspirin usage was higher among men (11.7% vs. 5.2%). Additionally, age-specific patterns emerged, indicating an inverse relationship between age and non-aspirin NSAIDs consumption, whereas aspirin use increased with advancing age. Notably, ibuprofen was the most commonly prescribed NSAIDs in this cohort study [1].

In a related investigation, Delaney et al. reported that over-the-counter (OTC) NSAIDs, particularly aspirin and ibuprofen, were more frequently utilized than prescribed alternatives. The study further revealed that the patterns of NSAIDs usage varied significantly based on socioeconomic status, behavioral factors, and ethnicity, highlighting disparities in access and utilization [13]. Furthermore, a study focusing on a northeastern Iranian population demonstrated that 19.3% of participants had consumed at least one NSAIDs. Diclofenac (49.21%), ibuprofen (28.6%), and naproxen (8%) were identified as the most frequently used NSAIDs within this population, reflecting regional prescription trends and preferences [14].

There are limited evidence exists on the comprehensive assessment of NSAIDs consumer characteristics in a general population. Most available studies focus on patients or medical prescriptions, leaving gaps in understanding NSAIDs usage patterns in broader cohorts. Considering the benefits and side effects of NSAIDs, alongside the potential for arbitrary use without medical prescriptions, it is crucial to investigate the prevalence of NSAIDs use and its associated factors in a general population within a cohort setting. Therefore, this study aims to determine the epidemiological and clinical profiles of NSAIDs users and the related factors in a large-scale, population-based cohort study.

## Methods

### Study design, setting and population

In this cross-sectional study, the data from enrolment phase of the Tabari cohort of Mazandaran University of Medical Sciences were used. The Tabari cohort study is part of a larger national cohort entitled Prospective Epidemiological Research Studies in Iran (PERSIAN) [15]. More details about the Tabari cohort are mentioned in the cohort profile [16, 17]. During the enrollment phase of the Tabari cohort, a total of 10,255 individuals aged 35 to 70 years were recruited from urban (*n* = 7,012) and mountainous (*n* = 3,243) regions of Sari, the capital of Mazandaran province in northern Iran, beside Caspian Sea.

### Inclusion and exclusion criteria

All individuals registered during the enrollment phase of the Tabari Cohort Study from June 2015 to November 2017 were included in the analysis, with the exception of cancer and kidney transplant patients, who were excluded due to their limited numbers. The inclusion criteria for the study required participants to be aged 35 to 70 years and residents of the study area. Exclusion criteria included individuals who declined to participate or were unable to communicate effectively due to disabilities such as deafness, blindness, muteness, or paralysis, particularly if these conditions prevented them from accessing the cohort center.

### Data collection and laboratory measurements

To ensure the privacy and confidentiality of participant information, data were collected anonymously without identifying individuals, using unique PERSIAN Cohort Identification (PCID) codes stored in an Excel format. Data collection in the Tabari Cohort Study encompassed a comprehensive range of measures, including a standardized questionnaire (covering general, medical, and nutritional information), along with blood pressure, pulse, anthropometric measurements, and biological samples (blood and urine). The details and design of the questionnaire are described in the methodology and cohort profile articles [15, 17].

Anthropometric indices, including height, weight, waist circumference, and hip circumference, were measured by trained personnel following standardized protocols. Height was recorded using a SECA 226 stadiometer (SECA, Hamburg, Germany), with participants instructed to stand barefoot, lean against the wall, and maintain proper alignment. Weight was measured using a SECA 755 hand scale (SECA, Hamburg, Germany). Patients were classified according to their Body Mass Index (BMI) as follows: BMI < 25 kg/m^2^ was considered normal, 25–29.9 kg/m^2^ as overweight, and ≥ 30 kg/m^2^ as obese [16]. Blood pressure was measured using validated methods outlined in the cohort protocol [15].

Blood samples were collected after 10–12 h of fasting and analyzed to determine fasting blood sugar, cholesterol, triglycerides (TG), and high-density lipoprotein (HDL) levels using an Auto Analyzer BT 1500 (Biotechnica, Italy). Complete blood count (CBC) analysis was performed using an alpha cell counter (Nihon Kohden, Tokyo, Japan). Additionally, morning urine samples were obtained in clean, chemical-free containers and processed for a complete urine analysis within two hours of collection.

The NSAIDs investigated in this study included aspirin, ibuprofen, naproxen, celecoxib, diclofenac, indomethacin, ketoprofen, ketorolac, mefenamic acid, meloxicam, piroxicam, and salicylate. Data on NSAIDs usage were obtained through self-reported information and a review of participants’ treatment booklets by trained interviewers.

### Definition of variables

Participants were classified as NSAID users if they had taken these drugs, both physician-prescribed and self-prescribed, in any form or route, including oral capsules, for at least 30 consecutive days routinely and regularly within a year [18].

A participant was identified as diabetic if they had a fasting blood sugar (FBS) level exceeding 126 mg/dL, had been formally diagnosed with diabetes by a physician, or were undergoing treatment with anti-diabetic medications. Hypertension was defined as a systolic blood pressure of 140 mmHg or higher, a diastolic blood pressure of 90 mmHg or higher, or a confirmed diagnosis of hypertension by a physician with concurrent treatment using antihypertensive medications.

The evaluation of chronic conditions such as cardiovascular disease, chronic headaches, depression and other psychiatric disorders, stroke, kidney stones, rheumatic diseases, osteoporosis, and back pain was based on self-reported physician-diagnosed history and the examination of participants’ medical records. Hyperlipidemia was determined through lipid profile analysis, while chronic kidney disease (CKD) was defined as a glomerular filtration rate (GFR) below 60 mL/min/1.73m^2^. Participants reporting a history of opium use were categorized as drug users, smokers were identified based on self-reported smoking history, and alcohol users were defined as those who reported a history of alcohol consumption.

In the analysis, marital status was categorized into two groups:"married" and "single". The "single" groupincluded individuals who were widowed, divorced, or had never married, and was combined into a single category due to the small sample size in each subgroup.

Socioeconomic status (SES) was assessed using principal component analysis (PCA), which integrated various economic and social variables, including domestic and international travel, book reading, computer and internet access, ownership of vehicles (car/motorcycle), home ownership versus tenancy, and possession of household appliances such as dishwashers, washing machines, freezers, and vacuum cleaners. Based on the PCA results, SES was categorized into five levels: Level I (weakest), Level II (poor), Level III (average), Level IV (good), and Level V (richest) [19].

### Statistical analysis

SPSS software version 27 was used for data analysis. Data description was expressed in percentage. Comparison between qualitative variables (grouped) was done using Chi-square test. In the univariate analysis, the odds ratios (ORs) for NSAID use, along with 95% confidence intervals (CIs), were calculated based on epidemiological and clinical variables using logistic regression. Additionally, two multivariable logistic regression models were implemented.

In the first model, the adjusted odds ratios (AORs) for NSAID use, along with 95% CIs, were estimated after adjusting for the following variables: gender, age, socioeconomic status, education level, marital status, body mass index (BMI), place of residence, employment status, alcohol use, substance use, smoking, diabetes, hypertension, coronary heart disease (CHD), dyslipidemia, CKD, stroke, kidney stones, rheumatic diseases, chronic headaches, depression and other psychiatric disorders, osteoporosis, and back pain.

In the second model, the adjusted odds ratios for NSAID use were calculated based on comorbidity. The comorbidity variable was constructed by combining 12 chronic conditions, including diabetes, hypertension, CHD, dyslipidemia, CKD, stroke, kidney stones, rheumatic diseases, chronic headaches, depression and other psychiatric disorders, osteoporosis, and back pain. This variable was subsequently categorized into eight levels: no comorbidity and one, two, three, four, five, six, and seven or more comorbidities. Finally, the adjusted odds ratios for NSAID use, along with 95% confidence intervals, were presented based on comorbidity, controlling for gender, socioeconomic status, place of residence, smoking, BMI, education years, marital status, age group, employment status, substance use, and alcohol consumption.

## Results

### Characteristics of participants

Of the 10,255 participants in the Tabari cohort, 9,939 individuals were included in the analysis. The majority of the participants were female (59.3%, *n* = 5,896) and urban residents (69.4%, *n* = 6,896). The distribution of participants by BMI was as follows: under/normal weight (BMI < 25), 23.9%; over weight (BMI 25–29.9), 42.5%; and obese (BMI ≥ 30), 33.6%.

The overall prevalence of NSAID use in the Tabari cohort population was 14.7% (*n* = 1,461). Among NSAID users, 30.6% (*n* = 447) reported use for 30 days to one year, while 69.4% (*n* = 1,015) reported use for more than one year. The most commonly reported NSAID was acetylsalicylic acid (ASA), used by 74.8% (*n* = 1,090) of users, followed by diclofenac (12.8%), celecoxib (8.1%), ibuprofen (5.4%), indomethacin 4.0%), Acetaminophen-Caffeine-Aspirin (ACA) (2.7%), mefenamic acid (1.3%), and piroxicam (0.6%). Regarding the number of NSAIDs used, 91.1% (*n* = 1,331) of users reported using one NSAID, 8.1% (*n* = 119) used two, 0.5% (*n* = 8) used three, and 0.2% (*n* = 3) used four different NSAIDs (Table 1).

**Table 1:** Patterns of NSAID consumption in the population

NSAID use characteristics	N (%)
Overall NSAID use (of total cohort)	1,461 (14.7%)
Use for 30 days to 1 year	446 (30.6%)
Use for more than 1 year	1015 (69.4%)
Most commonly used NSAIDs	
Acetylsalicylic acid (ASA)	1090 (74.8%)
Acetaminophen-Caffeine-Aspirin (ACA)	36 (2.7%)
Celecoxib	122 (8.1%)
Diclofenac	190 (12.8%)
Ibuprofen	81 (5.4%)
Indomethacin	61 (4.0%)
Mefenamic acid	15 (1.3%)
Piroxicam	10 (0.6%)
Number of NSAIDs used per participant	
One	1,331 (91.1%)
Two	119 (8.1%)
Three	8 (0.5%)
Four	3 (0.2%)

### Demographics and NSAIDs use

NSAID use did showed significant differences between genders (men 14.2% vs women 15.0%; *P* = 0.291). Prevalence increased steadily with age (35–39 y, 2.9%; 40–49 y, 7.6%; 50–59 y, 19.0%; 60–70 y, 30.5%; *P* < 0.001) and with BMI (< 25 kg/m^2^, 10.2%; 25–29.9 kg/m^2^, 14.0%; ≥ 30 kg/m^2^, 18.8%; *P* < 0.001). Single participants were also more likely to use NSAIDs than married participants (18.9% vs 14.3%; *P* = 0.001). Prevalence also varied by education, employment, and socioeconomic status (all *P* < 0.001) but not by place of residence (urban vs rural; *P* = 0.062) (Table 2).

**Table 2:** Characteristics of studied population by NASID consumption

Variables	Total; n	NSAID consumption		*P*-value (Chi-square test)
		User	Non-user	
Gender				
Male	4043	576 (14.2)	3467 (85.8)	0.291
Female	5896	885 (15)	5011 (85.0)	
Age group				
35–39	1599	47 (2.9)	1552 (97.1)	< 0.001
40–49	3372	257 (7.6)	3115 (92.4)	
50–59	3108	589 (19.0)	2519 (81.0)	
60–70	1860	568 (30.5)	1292 (69.5)	
Marital status				
Single	806	152 (18.9)	654 (81.1)	0.001
Married	9133	1309 (14.3)	7824 (85.7)	
Education Years				
University/College	2343	283 (12.1)	2060 (87.9)	< 0.001
9-12 years in school	2822	323 (11.4)	2499 (88.6)	
6–8 years in school	1094	150 (13.7)	944 (86.3)	
1–5 years in school	2250	381 (16.9)	1869 (83.1)	
No schooling	1430	324 (22.7)	1106 (77.3)	
BMI				
< 25	2379	243 (10.2)	2136 (89.8)	< 0.001
25–29.9	4221	591 (14.0)	3630 (86.0)	
≥ 30	3339	627 (18.8)	2712 (81.2)	
Place of residence				
Urban	6896	1044 (15.1)	5852 (84.9)	0.062
Mountainous	3043	417 (13.7)	2626 (86.3)	
Socioeconomic level				
1 (lowest)	1937	301 (15.5)	1636 (84.5)	< 0.001
2	1969	338 (17.2)	1631 (82.8)	
3	2005	301 (15.0)	1704 (85.0)	
4	2016	232 (11.5)	1784 (88.5)	
5 (highest)	2012	289 (14.4)	1723 (85.6)	
Employment status				
Employed	4294	450 (10.5)	3844 (89.5)	< 0.001
Housewife	4566	727 (15.9)	3839 (84.1)	
Unemployed	101	26 (25.7)	75 (74.3)	
Retired	978	258 (26.4)	720 (73.6)	
Smoker				
No	9034	1348 (14.9)	7686 (85.1)	0.049
Yes	905	113 (12.5)	792 (87.5)	
Alcohol use				
No	9148	1335 (14.6)	7813 (85.4)	0.309
Yes	791	126 (15.9)	665 (84.1)	
Drug abuse				
No	9339	1348 (14.4)	7991 (85.6)	0.003
Yes	600	113 (18.8)	487 (81.2)	
Diabetic				
No	8253	958 (11.6)	7295 (88.4)	< 0.001
Yes	1686	503 (29.8)	1183 (70.2)	
HTN				
No	7766	739 (9.5)	7027 (90.5)	< 0.001
Yes	2173	722 (33.2)	1451 (66.8)	
CHD				
No	9055	889 (9.8)	8166 (90.2)	< 0.001
Yes	884	572 (64.7)	312 (35.3)	
CKD				
No	7402	910 (12.3)	6492 (87.7)	< 0.001
Yes	2537	551 (21.7)	1986 (78.3)	
Dyslipidemia				
No	2305	205 (8.9)	2100 (91.1)	< 0.001
Yes	7634	1256 (16.5)	6378 (83.5)	
Depression and other psychiatric disorders				
No	8558	1187 (13.9)	7371 (86.1)	< 0.001
Yes	1381	274 (19.8)	1107 (80.2)	
Stroke				
No	9839	1406 (14.3)	8433 (85.7)	< 0.001
Yes	100	55 (55.0)	45 (45.0)	
Kidney Stone				
No	8331	1169 (14.0)	7162 (86.0)	< 0.001
Yes	1608	292 (18.2)	1316 (81.8)	
Rheumatic Disease				
No	9576	1365 (14.3)	8211 (85.7)	< 0.001
Yes	363	96 (26.4)	267 (73.6)	
Chronic Headaches				
No	8662	1241 (14.3)	7421 (85.7)	0.006
Yes	1277	220 (17.2)	1057 (82.8)	
Osteoporosis				
No	9252	1257 (13.6)	7995 (86.4)	< 0.001
Yes	687	204 (29.7)	483 (70.3)	
Back Pain				
No	5550	674 (12.1)	4876 (87.9)	< 0.001
Yes	4389	787 (17.9)	3602 (82.1)	

### Behavioral and comorbidity associations

Smoking was associated with significantly lower NSAIDs use compared to non-smoking (12.5% vs. 14.9%; *P* = 0.049). While alcohol consumption showed a slight but statistically insignificant increase in NSAIDs use (15.9% vs. 14.6%; *P* = 0.309), drug abuse was associated with significantly higher NSAIDs use (18.8% vs. 14.4%; *P* = 0.003) (Table 2).

NSAIDs use was significantly higher among participants with comorbid conditions, including diabetes, hypertension, CHD, CKD, dyslipidemia, depression and other psychiatric disorders, stroke, kidney stones, rheumatic disease, chronic headaches, osteoporosis, and back pain (all *P* < 0.001) (Table 2).

### Multivariable logistic regression analysis

The adjusted odds of NSAIDs use increased significantly with age, with highest odds ratio those aged 60–70 years (OR: 5.01 95% CI: 3.52–7.14) compared to the reference group (35–39 years). Similarly, obesity (BMI ≥ 30) was associated with a higher likelihood of NSAIDs use (OR: 1.40; 95% CI: 1.15–1.71) compared to a BMI < 25. Other significant predictors included urban residence, unemployment, and drug abuse (Table 3).

**Table 3:** Factors associated with prevalence of NASID in Tabari cohort study

Variables	Crud prevalence of NASID (95% CI)	Adjusted prevalence of NASID (95% CI)
Gender		
Male	Ref	Ref
Female	1.06 (0.95–1.16)	0.82 (0.64–1.04)
Age group		
35–39	Ref	Ref
40–49	2.72 (1.98–3.74)	2.00 (1.44–2.78)
50–59	7.72 (5.70–10.46)	3.61 (2.60–5.02)
60–70	14.52 (10.68–19.72)	5.01 (3.52–7.14)
Marital status		
Married	Ref	Ref
Single	1.39 (1.15–1.67)	1.20 (0.95–1.52)
Education Years		
University/College	Ref	Ref
9-12 years in school	0.48 (0.94–1.12)	0.87 (0.71–1.07)
6–8 years in school	1.16 (0.93–1.43)	0.80 (0.61–1.06)
1–5 years in school	1.48 (1.26–1.75)	1.01 (0.78–1.30)
No schooling	2.13 (1.79–2.54)	0.93 (0.68–1.26)
BMI		
< 25	Ref	Ref
25–29.9	1.43 (1.22–1.68)	1.16 (0.96–1.40)
≥30	2.03 (1.73–2.38)	1.40 (1.15–1.71)
Place of residence		
Mountainous	Ref	Ref
Urban	1.12 (0.99–1.27)	1.71 (1.41–2.08)
Socioeconomic level		
V (highest)	Ref	Ref
IV	0.77 (0.64–0.93)	0.92 (0.74–1.15)
III	1.05 (0.88–1.25)	1.06 (0.85–1.33)
II	1.24 (1.04–1.47)	1.20 (0.95–1.52)
I (lowest)	1.10 (0.92–1.31)	1.08 (0.82–1.43)
Employment status		
Employed	Ref	Ref
Housewife	1.62 (1.43–1.84)	1.17 (0.92–1.48)
Unemployed	2.96 (1.88–4.67)	1.84 (1.06–3.21)
Retired	3.06 (2.58–3.64)	1.18 (0.95–1.47)
Smoker		
No	Ref	Ref
Yes	0.81 (0.66–1.00)	0.99 (0.76–1.30)
Alcohol use		
No	Ref	Ref
Yes	1.11 (0.91–1.35)	1.13 (0.86–1.47)
Drug abuse		
No	Ref	Ref
Yes	1.38 (1.11–1.70)	1.34 (1.01–1.79)
Diabetic		
No	Ref	Ref
Yes	3.24 (2.86–3.67)	1.66 (1.43–1.93)
HTN		
No	Ref	Ref
Yes	4.73 (4.21–5.32)	2.34 (2.03–2.69)
CHD		
No	Ref	Ref
Yes	16.84 (14.43–19.65)	10.41 (8.78–12.34)
CKD		
No	Ref	Ref
Yes	1.98 (1.76–2.22)	1.14 (0.99–1.32)
Dyslipidemia		
No	Ref	Ref
Yes	2.02 (1.73–2.36)	1.32 (1.10–1.58)
Depression and other psychiatric disorders		
No	Ref	Ref
Yes	1.54 (1.33–1.78)	1.01 (0.84–1.21)
Stroke		
No	Ref	Ref
Yes	7.33 (4.92–10.91)	3.40 (2.10–5.51)
Kidney Stone		
No	Ref	Ref
Yes	1.36 (1.18–1.56)	0.90 (0.76–1.07)
Rheumatic Disease		
No	Ref	Ref
Yes	2.16 (1.70–2.75)	1.30 (0.97–1.75)
Chronic Headaches		
No	Ref	Ref
Yes	1.24 (1.06–1.46)	1.24 (1.02–1.50)
Osteoporosis		
No	Ref	Ref
Yes	2.69 (2.26–3.20)	1.34 (1.07–1.68)
Back Pain		
No	Ref	Ref
Yes	1.58 (1.41–1.77)	1.21 (1.06–1.39)

Comorbid conditions were strong predictors of NSAIDs use with highest adjusted odds ratios in individuals with CHD (OR: 10.41; 95% CI: 8.78–12.34), stroke (OR: 3.40; 95% CI: 2.10–5.51) and hypertension (OR: 2.34; 95% CI: 2.03–2.69. No significant associations were found for gender, marital status, education, socioeconomic status, smoking, alcohol use, CKD, depression, kidney stones, or rheumatic disease (Table 3).

### NSAID use and comorbidity levels

The prevalence of NSAIDs use significantly increased with the number of comorbid conditions: 1.9% for individuals without comorbidities, 3.9% for those with one condition, 8.7% with two, 16.4% with three, 28.8% with four, 35.0% with five, 42.8% with six, and 55.1% with seven or more comorbidities. After adjusting for gender, socioeconomic status, place of residence, smoking, BMI, education, marital status, age group, employment status, drug abuse, and alcohol consumption, the adjusted odds ratios for NSAIDs use increased progressively with the number of comorbidities: (OR: 2.06, 95% CI: 1.14–3.72) for one condition, (OR: 4.27, 95% CI: 2.41–7.56) for two, (OR: 7.54, 95% CI: 4.26–13.34) for three, (OR: 14.14, 95% CI: 7.97–25.09) for four, (OR: 17.58, 95% CI: 9.79–31.57) for five, (OR: 21.72, 95% CI: 11.78–40.03) for six, and (OR: 36.35, 95% CI: 18.93–69.80) for seven or more comorbidities (P for trend < 0.001) (Table 4).

**Table 4:** Comorbidity and prevalence of NASID in Tabari Cohort study

Number of comorbidities	Total; n	Use NASID		Crud prevalence of NASID (95% CI)	Adjusted prevalence of NASID (95% CI)*
		n	%		
0	680	13	1.9	Ref	Ref
1	2308	91	3.9	2.11 (1.17–3.79)	2.06 (1.14–3.72)
2	2606	227	8.7	4.90 (2.78–8.62)	4.27 (2.41–7.56)
3	1986	326	16.4	10.08 (5.75–17.67)	7.54 (4.26–13.34)
4	1242	358	28.8	20.78 (11.84–36.46)	14.14 (7.97–25.09)
5	662	232	35.0	27.68 (15.63–49.03)	17.58 (9.79–31.57)
6	299	128	42.8	38.41 (21.19–69.61)	21.72 (11.78–40.03)
≥ 7	156	86	55.1	63.03 (33.47–118.72)	36.35 (18.93–69.80)
P for trend				< 0.001	< 0.001

^*^Adjusted for gender, socioeconomic level, place of residence, smoking, BMI, Education Years, marital status , age group, employment status, drug abuse and alcohol use

## Discussion

This study examined the prevalence of NSAIDs use and its associated factors in individuals aged 35 to 70 years from the Tabari Cohort. The findings revealed that 14.7% of participants in the Tabari Cohort reported using at least one of the eight NSAIDs studied (ASA, ACA, celecoxib, diclofenac, ibuprofen, indomethacin, mefenamic acid, piroxicam). The odds of NSAID use significantly increased with the number of comorbidities. The results of multivariable logistic regression analysis also demonstrated significant associations between NSAIDs use and several demographic and clinical factors.

The observed NSAIDs consumption rate in our study (14.7%) is lower than that reported in northeastern Iran (19.3%) [14]. Comparing the prevalence in global level, across Europe, prevalence ranges from 18% in Italy [20] to 30% in Spain [1], broadly higher than our 14.7% prevalence in Tabari cohort. However, our findings closely rely with findings from study in Malaysia [21] reporting the overall prevalence of 14.2% NSAIDs use among adults. These differences may be attributed to several factors, including regional prescription practices, availability of over-the-counter medications, cultural attitudes toward self-medication, healthcare access, and differences in comorbidity prevalence.

The Tabari Cohort finding that NSAIDs use increases significantly with age mirrors evidence from other populations. Older age groups often have a higher prevalence of conditions that drive NSAIDs use, such as osteoarthritis and chronic pain [22]. The findings from a U.S. study using NHANES data, highlighted that participants over 60 years were significantly more likely to be regular NSAIDs users [23]. In another population-based study in Italy, increasing age was associated with more frequent and increased risk of chronic use of NSAIDs [20].

Obesity was also significantly associated with higher odds of NSAIDs use in our study. Similarly in a U.S population study, the BMI > 30 were more likely to be regular users of NSAIDs than BMI < 25 [23]. This condition is a well-known contributor to chronic musculoskeletal pain, particularly osteoarthritis through joint stress and also local and systemic inflammation mechanisms [24] and has been shown to be significantly associated with persistent pain conditions [25], which often necessities the increased use of NSAIDs for pain management.

Urban residence was also significantly associated with NSAIDs use in our study. Similarly, a study in Australia found that urban general practitioners (GPs) were more likely to feel pressured by patients to prescribe NSAIDs, due to patients' greater awareness and requests for these medications [26]. In another US study, rural residents were more likely to use non-medication therapies for chronic pain conditions [27]. Urban populations tend to have greater access to healthcare providers and pharmacies, which facilitates more frequent medical consultations and higher prescription rates for NSAIDs. In contrast, rural residents often face significant barriers such as longer distances to health facilities and fewer healthcare resources, limiting their access to professional medical care [28].

Our study demonstrated a significant association between drug abuse and NSAID consumption. While direct prevalence data is limited, drug abusers has been reported chronic pain and pain-related dysfunction which might lead to self-prescription to mediate their pain [29].

Unemployed individuals also had significantly higher odd of NSAID consumption in our study. Similarly, a study in Danish population showed unemployment was significantly associated with NSAID initiation and continuing use [30]. Unemployment is associated with increased rate of comorbid condition [31] and poor health-care access [32] prompting reliance on easily accessible over-the-counter drugs like NSAIDs for symptom management.

In this study various comorbid conditions including diabetes, hypertension, stroke, CHD, dyslipidemia, chronic back pain and osteoporosis have been significantly associated with NSAIDs use and the magnitude increased progressively with the number of comorbidities.

The interaction between diabetes and NSAIDs use is multifaceted, with studies highlighting both potential benefits and risks. NSAIDs may offer metabolic advantages, such as reducing systemic inflammation and improving insulin sensitivity, which could help in diabetes prevention or management [33, 34]. However, these benefits are offset by risks, including an increased likelihood of adverse effects such as acute kidney injury (AKI) and cardiovascular events in diabetic populations, especially when combined with other medications like renin–angiotensin–aldosterone system blockers [35]. Furthermore, chronic NSAIDs use in diabetic patients impose significant risks for gastrointestinal bleeding and cardiovascular events [36].

Early studies showed the significant effect of NSAIDs use as a risk factor for hypertension [37, 38]. Further studies also showed that NSAIDs users having 2 mmHg increase in their systolic blood pressure [39]. Moreover, NSAIDs can antagonize antihypertensive therapies, particularly Angiotensin Converting Enzyme (ACE) inhibitors and beta-blockers, leading to less effective blood pressure control [40]. The use of NSAIDs has been consistently associated with increased cardiovascular risks, particularly in patients with CHD or those at risk for stroke. NSAIDs use in individuals with stable atherothrombosis is linked to a higher risk of myocardial infarction, stroke, and heart failure-related hospitalizations [41]. Additionally chronic NSAIDs use in patients with hypertension and CAD increased the risk cardiovascular mortality in long-term [42]. NSAIDs, particularly COX-2 inhibitors and certain nonselective agents like diclofenac and meloxicam, are associated with an elevated risk of hemorrhagic stroke, with relative risk increases ranging from 1.27 to 1.48 in observational studies [43]. NSAIDs use in patients with multiple comorbidities is associated with increased risks of adverse outcomes, requiring careful consideration and monitoring. Studies highlight that NSAIDs are frequently prescribed for pain and inflammation in individuals with conditions like hypertension, diabetes, cardiovascular diseases, and chronic kidney disease, despite these conditions heightening the risk of NSAID-related complications [44].

Given the risks associated with NSAIDs use, effective management strategies, including alternative therapies and co-prescription of gastroprotective agents, are critical to reducing these risks while maintaining pain relief [44]. In high-risk patients with comorbidities other alternative options including acetaminophen, physiotherapy, or non-pharmacologic therapies for pain management could also be considered [45].

Our findings are based on a specific geographic and demographic population within the Tabari Cohort, which may limit the generalizability of the results to other populations with different health system structures, prescribing practices, and cultural contexts.

One limitation of the present study is the reliance on self-reported data by participants when providing documentation related to their medication use to the interviewers. Therefore, there is a possibility that some participants either did not present certain documents or evidence regarding their medication use or did not have access to them. This issue could potentially lead to underreporting of non-steroidal drug use in the study. Thus, caution is advised when interpreting the results. It should be noted that this limitation was acknowledged at the outset of the study, and during data collection and recording, measures were implemented to minimize this limitation. Another limitation of the study is the inability to distinguish between self-prescribed medication use and physician-prescribed medication. It is important to note that the strong association observed between CHD and NSAID use may, in part, reflect the widespread clinical recommendation of low-dose aspirin for cardiovascular prophylaxis. Due to data limitations, we could not differentiate between prophylactic and therapeutic use of aspirin. This may lead to an overestimation of NSAID use among CHD patients and should be considered when interpreting the results. Due to cross-sectional nature of the study, the causal relationship between variables could not be determined. Another limitation of this study is that multicollinearity among variables, particularly overlapping comorbidities, was not formally assessed using statistical diagnostics such as variance inflation factors (VIFs). This may have influenced the precision of estimated associations in the multivariable models.

One of the prominent strengths of the present study is its large sample size, which enabled the evaluation of numerous variables in predicting non-steroidal drug use through a multiple regression model. Additionally, the use of data recorded in the Tabari Cohort Study, which are highly reliable, provides the necessary foundation for generalizing the results.

## Conclusion

This study highlights key predictors of NSAID use including age, obesity, urban living, and comorbidities and emphasizes their application in clinical and public health settings. The findings support targeted interventions, safer prescribing practices, and public education to reduce risks, particularly in vulnerable populations with multiple chronic conditions. These insights can inform healthcare policies and promote more effective, individualized pain management strategies.

## Data Availability

No datasets were generated or analysed during the current study.

## Abbreviations

NSAIDs: Non-steroidal anti-inflammatory drugs
DM: Diabetes Mellitus
HTN: Hypertension
CHD: Coronary Heart Disease
CKD: Chronic kidney disease
BMI: Body Mass Index
HDL: High-density Lipoprotein
TG: Triglycerides
TC: Total cholesterol
ASA: Acetylsalicylic acid
ACA: Acetaminophen-Caffeine-Aspirin
COX: Cyclooxygenase
FBS: Fasting blood sugar
GFR: Glomerular filtration rate
